# Extracellular Electron Transfer May Be an Overlooked Contribution to Pelagic Respiration in Humic-Rich Freshwater Lakes

**DOI:** 10.1128/mSphere.00436-18

**Published:** 2019-01-23

**Authors:** Shaomei He, Maximilian P. Lau, Alexandra M. Linz, Eric E. Roden, Katherine D. McMahon

**Affiliations:** a Department of Bacteriology, University of Wisconsin—Madison, Madison, Wisconsin, USA; b Department of Geoscience, University of Wisconsin—Madison, Madison, Wisconsin, USA; c Département des Sciences Biologiques, Université du Québec à Montréal (UQAM), Montréal, Quebec, Canada; d Department of Civil and Environmental Engineering, University of Wisconsin—Madison, Madison, Wisconsin, USA; University of Minnesota

**Keywords:** Cyc2, extracellular electron transfer, EET, humic lake, humic substances, HS, iron, Fe, multiheme cytochrome *c*, MHC, porin-cytochrome *c* complex, PCC, redox cycling

## Abstract

Humic lakes and ponds receive large amounts of terrestrial carbon and are important components of the global carbon cycle, yet how their redox cycling influences the carbon budget is not fully understood. Here we compared metagenomes obtained from a humic bog and a clear-water eutrophic lake and found a much larger number of genes that might be involved in extracellular electron transfer (EET) for iron redox reactions and humic substance (HS) reduction in the bog than in the clear-water lake, consistent with the much higher iron and HS levels in the bog. These genes were particularly rich in the bog’s anoxic hypolimnion and were found in diverse bacterial lineages, some of which are relatives of known iron oxidizers or iron-HS reducers. We hypothesize that HS may be a previously overlooked electron acceptor and that EET-enabled redox cycling may be important in pelagic respiration and greenhouse gas budget in humic-rich freshwater lakes.

## Abstract (precis)

Humic lakes and ponds receive large amounts of terrestrial carbon and are important components of the global carbon cycle, yet how their redox cycling influences the carbon budget is not fully understood. Here we compared metagenomes obtained from a humic bog and a clear-water eutrophic lake and found a much larger number of genes that might be involved in extracellular electron transfer (EET) for iron redox reactions and humic substance (HS) reduction in the bog than in the clear-water lake, consistent with the much higher iron and HS levels in the bog.

## OPINION/HYPOTHESIS

Inland lakes receive allochthonous carbon (C) fixed in their catchment areas, and they play an important role in the cycling of terrestrial C and affect global C budgets. In the past decades, many northern freshwater lakes have been experiencing an increase in water color known as “browning,” and this trend may continue with changes in precipitation patterns and atmospheric deposition chemistry (1). The browning water may pose environmental concerns and impact freshwater functions (e.g., increasing drinking water treatment cost, decreasing primary productivity, and increasing anoxia due to decreased light penetration, affecting global-scale C cycling). A leading factor contributing to the browning process is the increasing inputs of allochthonous dissolved organic C (DOC) (2). A major component of terrestrially derived allochthonous DOC in freshwater is humic substances (HS), which are heterogeneous mixtures of naturally occurring recalcitrant organic carbon derived from plant and animal decay. Another factor contributing to surface water browning is increasing iron (Fe) inputs, which were positively correlated to the increasing organic C inputs (3, 4). This correlation may partly be due to the complexation of Fe by organic matter, in particular HS, as the complexation may increase Fe leaching from catchment soil and maintain Fe in the water column instead of removing it by sedimentation within the receiving water body (4).

Increased inputs of HS and Fe have the potential to impact overall lake metabolism in two basic ways. First, HS and the more-labile low-molecular-weight C derived from HS photodegradation serve as important C sources for heterotrophic respiration in humic lakes (5). Second, both Fe and HS can serve as electron acceptors for anaerobic respiration in sediments and anoxic hypolimnetic waters. Microbially catalyzed Fe (and Mn) reduction is a well-known process in stratified lakes, and Fe was recently shown to undergo rapid “cryptic” cycling in lakes, potentially mediating 10% of total carbon turnover despite Fe being present at very low bulk concentrations (6). HS can also serve as an electron acceptor through the reduction of their quinone moieties, and their electron-accepting capacity is fully regenerable under recurrent oxic/anoxic transitions (7). However, most prior research on the electron-accepting capacity of HS considered the impact on C cycling in wetlands, sediments, and soils, rather than truly pelagic ecosystems (7, 8). Recently, a study on a humic lake showed that native organic matter with more oxidized quinone moieties and therefore higher electron-accepting capacity favored freshwater bacterial growth and production under anoxic conditions and further suggested organic matter as an important electron acceptor in stratified lakes with oxycline fluctuations (9). Despite this, the role of HS as an electron acceptor in freshwater lakes has not been widely appreciated, particularly in relation to the potential for water column C metabolism coupled to cryptic cycling, as has been demonstrated for Fe (6).

Theoretically, if HS are used to respire organic C, this has the potential to lower methane emissions from lakes. The reduction potential distribution in HS suggests HS reduction to be thermodynamically more favorable than methanogenesis in anoxic waters (7). As the resulting competitive mitigation of methanogenesis was observed in peat bogs and peat soils (10), a similar process is expected for pelagic respiration in lakes. Therefore, we judge it timely to further explore the contribution of HS and Fe reduction to pelagic respiration in freshwater lakes.

In humic lakes, light does not penetrate deep into the water column due to its absorbance by HS and Fe. Therefore, humic lakes generally have a shallower phototrophic (and therefore oxygenated) zone than clear-water lakes during stratification, leaving a larger proportion of the water column under anoxic conditions. Due to this redox distribution and their high concentrations, HS and Fe may become important terminal electron acceptors in humic lakes. Thus, here we present the hypothesis that HS and Fe redox cycling is more significant in humic lakes than in clear-water lakes and that these redox processes may influence ecosystem-level C budgets (i.e., overall lake metabolism). As a preliminary examination of this hypothesis, we studied two contrasting temperate lakes, including a small humic lake, Trout Bog, in which the DOC is highly aromatic and primarily of terrestrial origin (11), and a large eutrophic clear-water lake, Lake Mendota, which has much lower concentrations of HS and Fe than Trout Bog, with most of its DOC being produced in-lake via photosynthesis. Detailed lake characteristics are listed in Table S1 in the supplemental material, and representative depth profiles of temperature, dissolved oxygen, and total soluble Fe during summer stratification are shown in Fig. S1. Three combined assemblies of time series metagenome libraries previously obtained from Lake Mendota epilimnion (ME), Trout Bog epilimnion (TE), and Trout Bog hypolimnion (TH), respectively, and over 200 metagenome-assembled genomes (MAGs) were recovered from these combined assemblies (12, 13). We examined these metagenomes and MAGs to identify genes involved in HS and Fe redox processes to compare their distributions in the two contrasting lakes.

10.1128/mSphere.00436-18.2 FIG S1 Representative depth profiles of temperature (a and b), dissolved oxygen (c and d), and total soluble Fe (e and f) for Lake Mendota and Trout Bog, respectively, during summer stratification. Trout Bog and Lake Mendota temperature and dissolved oxygen profiles were collected on 10 July 2007 and 1 August 2014, respectively. The Fe data were collected in August 2012 for both lakes. Data were retrieved from the North Temperate Lakes Long Term Ecological Research site database available at https://lter.limnology.wisc.edu/. Metadata describing how the data were collected are also available on the site. Download FIG S1, PDF file, 0.05 MB. Copyright © 2019 He et al. 2019 He et al. This content is distributed under the terms of the Creative Commons Attribution 4.0 International license.

10.1128/mSphere.00436-18.3 TABLE S1 Lake characteristics. Download Table S1, XLSX file, 0.03 MB. Copyright © 2019 He et al. 2019 He et al. This content is distributed under the terms of the Creative Commons Attribution 4.0 International license.

Due to the high molecular weight of HS and the poor solubility of Fe(III), these electron acceptors are reduced extracellularly via a process called extracellular electron transfer (EET). The reduced HS and Fe can be abiotically reoxidized by oxygen under oxic conditions. In addition, biological Fe(II) oxidation may occur, and this employs EET due to the poor solubility of the reaction product, Fe(III). One form of oxidoreductase in Fe redox EET processes involves outer surface proteins, such as Cyc2, a monoheme cytochrome *c* (Cyt *c*) typically found in Fe(II) oxidizers (14), and multiheme *c*-type cytochromes (MHCs) in Fe(III) reducers (15). Another form of EET oxidoreductase forms a porin-cytochrome *c* protein complex (PCC), in which the oxidoreductase, usually an MHC, is secreted to the periplasm and embedded into a porin on the outer membrane to form the EET conduit (16). Most Fe(III) reducers can also reduce HS (17) and probably use the same EET systems to transfer electrons to HS. For example, in Geobacter sulfurreducens, a number of outer membrane MHCs that are important in the reduction of Fe(III) are able to reduce extracellular antraquinone-2,6-disulfonate (AQDS; a humic acid analogue) and HS (18), and in Shewanella oneidensis, the porin and periplasmic MHC components of its Fe(III)-reducing PCC are essential for AQDS and HS reduction (19, 20). These findings suggest that reduction of the quinone moieties in HS is a nonspecific redox process by EET systems.

In this study, we searched for putative EET genes (including genes encoding PCC, outer surface MHCs not associated with PCC, and Cyc2) in MAGs and metagenomes to examine if these genes are indeed more abundant in a humic bog than in a clear-water lake. Method details on the identification and quantification of putative EET genes are described in Text S1 in the supplemental material. All (meta)genome data are publicly available at JGI’s Integrated Microbial Genomes & Microbiomes (IMG/M; https://img.jgi.doe.gov/m). The IMG/M identifiers (IDs) for the ME, TE, and TH metagenomes are 3300002835, 3300000439, and 3300000553, respectively, and the IMG IDs for putative EET gene-containing MAGs (together with details on these MAGs and putative EET genes in the three metagenomes) are listed in Tables S2 and S3. A more comprehensive analysis of the full MAG data set is published elsewhere (13).

10.1128/mSphere.00436-18.1 TEXT S1 Supplemental materials and methods. Download Text S1, DOCX file, 0.03 MB. Copyright © 2019 He et al. 2019 He et al. This content is distributed under the terms of the Creative Commons Attribution 4.0 International license.

10.1128/mSphere.00436-18.4 TABLE S2 List of MHCs that might be involved in EET in the metagenomes. Download Table S2, XLSX file, 0.01 MB. Copyright © 2019 He et al. 2019 He et al. This content is distributed under the terms of the Creative Commons Attribution 4.0 International license.

10.1128/mSphere.00436-18.5 TABLE S3 List of Cyc2-like genes in the metagenomes. Download Table S3, XLSX file, 0.04 MB. Copyright © 2019 He et al. 2019 He et al. This content is distributed under the terms of the Creative Commons Attribution 4.0 International license.

**FIG 1:**
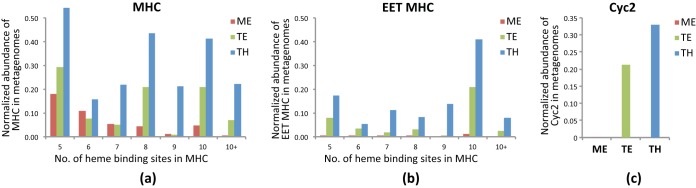
Normalized abundances of multiheme *c*-type cytochromes (MHCs) (a), MHCs with putative EET functions (i.e., MHCs in PCC and outer surface MHCs not associated with PCC) (b), and Cyc2 homologs (c) found in metagenomes obtained from Lake Mendota’s epilimnion (ME), and Trout Bog’s epilimnion (TE) and hypolimnion (TH), respectively. (a and b) Normalized abundance was reported for MHCs with 5 to 10 and >10 heme binding sites, respectively. The normalized abundance was obtained by mapping metagenome reads to assembled contigs, and the read coverage was then normalized by the average read coverage of single-copy conserved bacterial housekeeping genes in the same metagenome. See Text S1 in the supplemental material for details on the calculation of normalized abundance.

MHCs are important components of EET systems involved in Fe redox reactions and HS reduction. In particular, MHCs with large numbers of hemes may be able to form molecular “wires” for conducting electrons from the periplasmic space across the outer membrane (21). We therefore estimated the normalized abundance of MHCs with at least five heme-binding sites in the metagenomes. In general, TH had the highest abundance of MHCs, followed by TE and ME, and such differences were even more pronounced for MHCs with at least eight heme-binding sites (Fig. 1A). Some of these MHCs are components of other redox enzyme complexes, such as the pentaheme and hexaheme MHCs in alternative complex III and octaheme MHCs in tetrathionate reductases and hydroxylamine oxidoreductases. Putative EET MHC components (i.e., MHCs in PCC and outer surface MHCs not associated with PCC, as listed in Table S2) were much more frequently found in MHCs with large numbers of heme binding sites (e.g., >9), and these putative EET genes were more abundant in TH than in TE and nearly absent in the ME metagenome (Fig. 1B). This may indicate that MHC-based EET potential was more significant in the anoxic layer than in the oxic layer of the humic bog and was minimal in the oxic layer of the clear-water lake with low Fe and HS concentrations. Notably, the largest number of heme-binding sites (i.e., 51) was found in an MHC component of a putative PCC, encoded in an unbinned contig in the TE metagenome (Table S2).

### Porin-PCC genes.

The best-studied PCC system, MtrABC (consisting of a porin, a periplasmic decaheme Cyt *c*, and an extracellular decaheme Cyt *c*), was first identified in S. oneidensis as being essential for Fe(III) reduction (16). Their homologous PCCs, PioAB and MtoAB, which lack the extracellular MHC component, were suggested to be involved in Fe(II) oxidation in the phototrophic Rhodopseudomonas palustris strain TIE-1 (22) and the microaerophilic Fe(II) oxidizers in the family of *Gallionellaceae* (23), respectively. The more recently discovered PCC proteins in G. sulfurreducens are not homologous to MtrABC but are also encoded in operons with genes encoding a porin (OmbB), a periplasmic octaheme Cyt *c* (OmaB), and an outer membrane dodecaheme Cyt *c* (OmcB) (24). This suggests that multiple PCC systems evolved independently and may provide a clue to search for new types of PCC by examining genome-level organization. For example, putative novel PCC genes not homologous to previously identified PCCs were found in some Fe(II) oxidizer genomes by searching for the unique genetic organization of porin- and periplasmic MHC-coding genes (25).

**FIG 2:**
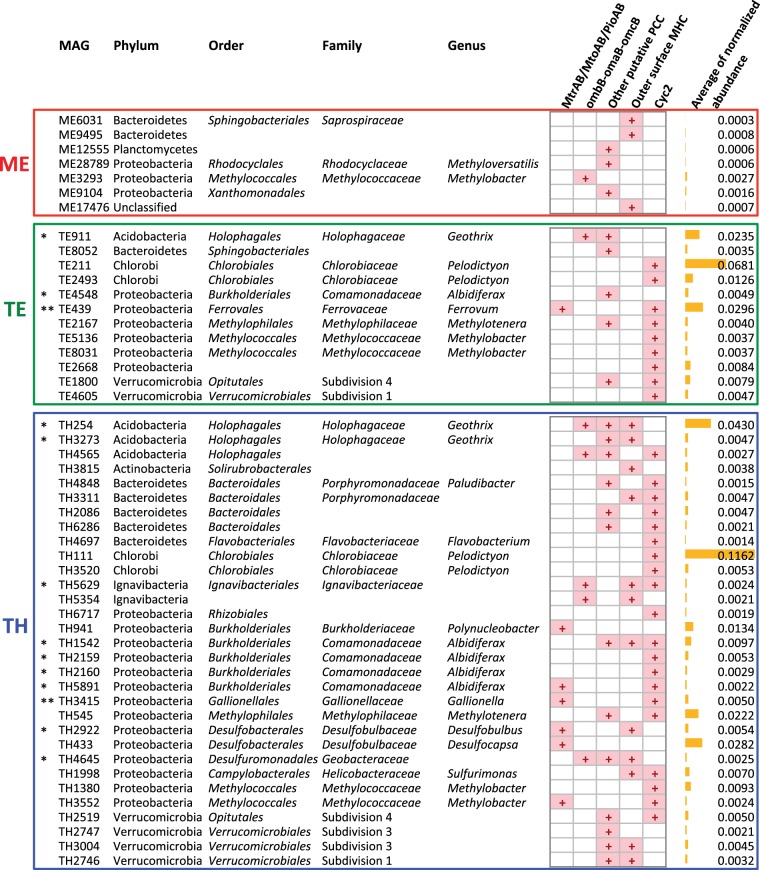
Occurrence of putative EET genes in MAGs and the normalized abundance of EET genes in each MAG as measured by mapping reads to assembled contigs for read coverage and normalizing by the average coverage of single-copy conserved bacterial housekeeping genes in the metagenome (see Text S1 in the supplemental material for details). If multiple EET genes were identified in one MAG, their normalized abundances were very comparable since they were from the same MAG, and thus, the average normalized abundance from all EET genes in that MAG was reported. Therefore, the normalized abundance reported in this figure also indicates the significance of populations represented by these MAGs in the lake. *, MAGs with Fe(III)-reducing relatives; **, MAGs with Fe(II)-oxidizing relatives; +, the presence of putative EET genes.

Nearly all MtrAB/MtoAB/PioAB homologs were recovered in Trout Bog and mostly from TH (Table S2). They are present in MAGs affiliated with the proteobacteria, including Fe(II)-oxidizing *Gallionella* and *Ferrovum*, Fe(III)-reducing *Albidiferax*, Fe(III)- and AQDS-reducing *Desulfobulbus*, and genera not known for EET, such as *Polynucleobacter*, *Desulfocapsa*, and *Methylobacter* (Fig. 2). Interestingly, among the 46 *Polynucleobacter* genomes available at IMG/M (https://img.jgi.doe.gov/m), MtrAB/MtoAB/PioAB homologs were found only in *Polynucleobacter* organisms recovered from a wetland and two humic lakes (including Trout Bog and Lake Grosse Fuchskuhle located in Brandenburg, Germany), suggesting that this PCC might be an acquired trait of some *Polynucleobacter* spp. adapting to humic-rich environments.

Homologs of another studied PCC (represented by OmbB-OmaB-OmcB in *Geobacter* spp.) were present in MAGs affiliated with relatives of known Fe(III) (and HS) reducers, including *Geothrix*, *Ignavibacteriaceae*, and *Geobacteraceae*, as well as in *Methylobacter* (Fig. 2).

Based on the unique genetic organization of PCC-encoding genes, we found a number of putative PCCs that do not share a significant sequence homology with known PCCs, probably representing novel PCC types. These putative PCC genes were present in Fe(III) (and HS) reducers (*Geothrix*, *Albidiferax*, and *Geobacteraceae*) and bacteria not known for EET, including *Methylotenera*, *Methylobacter*, *Methyloversatilis*, and a number of *Bacteroidetes* and *Verrucomicrobia* (Fig. 2). Among them, members of the phylum *Verrucomicrobia* with putative PCC genes were previously found in humic-rich environments, such as soils and lake sediment, in addition to the *Verrucomicrobia* MAGs from Trout Bog (25).

### Outer surface MHCs not associated with the PCC.

Outer surface MHCs that are not PCC components may also be involved in EET. Examples include OmcE, OmcS, and OmcZ in G. sulfurreducens (15), outer surface MHCs in Gram-positive Fe(III)- and AQDS-reducing Firmicutes (26), and MHCs in deltaproteobacterial sulfate-reducing bacteria that may be responsible for EET with its anaerobic CH_4_-oxidizing archaeal syntrophic partner (27).

Here, we found a number of non-PCC-associated outer surface MHCs in the metagenomes (Table S2) and MAGs, including Fe(III) (and HS)-reducing taxa (*Albidiferax*, *Geothrix*, *Desulfobulbus*, *Ignavibacteriaceae*, and *Geobacteraceae*) and several members in the *Bacteroidetes* and *Verrucomicrobia* phyla (Fig. 2). In particular, seven genes predicted to encode MHCs located on the cell wall were found in a Gram-positive actinobacterial MAG classified to *Solirubrobacterales* from TH, and four of these genes are located in the same gene cluster with up to 15 heme-binding sites in a single MHC (Table S2), probably involved in electron transfer on the cell wall.

### Cyc2.

Cyc2 is an outer membrane *c*-type cytochrome with one heme-binding motif in the N terminus and a predicted porin structure at the C terminus. Cyc2 was originally identified as the Fe(II) oxidase in acidophilic Acidithiobacillus ferrooxidans (14), with distant homologs later found in neutrophilic microaerobic *Mariprofundus* spp. and some other neutrophilic Fe(II) oxidizers (see the review by He et al. [25]).

As with EET MHC genes, the normalized abundance of total Cyc2-like genes was much higher in the TH than in the TE metagenome, and Cyc2-like genes were largely absent in the ME metagenome (Fig. 1C). Cyc2 homologs were present in 29 MAGs exclusively from Trout Bog (Table S3 in the supplemental material), including relatives of Fe(II)-oxidizing genera (*Ferrovum* and *Gallionella*) and Fe(III)-reducing taxa (*Ignavibacteriaceae* and *Albidiferax*), as well as bacteria not known for EET, including *Methylotenera*, *Methylobacter*, *Pelodictyon*, and members of the *Bacteroidetes* and *Verrucomicrobia* (Fig. 2).

### Electron-accepting capacity of Trout Bog water.

In the current study, we measured the electron-accepting capacity of HS in the epilimnion and hypolimnion water of Trout Bog according to the method of Kappler et al. (8), and the electron-accepting capacities of the epilimnion and hypolimnion water were 0.115 and 0.128 mM, respectively (see Text S1 for the determination of lake water electron-accepting capacity).

With the ongoing brownification of surface water due to increasing inputs of terrestrial C and Fe on a large scale, elucidating the roles and contribution of HS and Fe in redox and C cycling becomes even more relevant to C budgets at an ecosystem level. Here, we inspected EET genes/organisms potentially involved in HS and Fe redox processes in two freshwater lakes with contrasting HS and Fe levels to examine if these genes/organisms were more abundant in the humic lake, particularly in its anoxic layer. All together, a total of 103, 36, and 66 MAGs were recovered from the ME, TE, and TH metagenomes, respectively. Among them, putative EET genes were found in 7, 12, and 31 MAGs from ME, TE, and TH, respectively (Fig. 2). Therefore, a larger fraction of MAGs might encode the EET function in Trout Bog, especially in its hypolimnion, than in Lake Mendota. This, together with the normalized abundance of putative EET genes in the three metagenomes (Fig. 1), suggests that the genetic potential of EET was more significant in the anoxic layer than in the oxic layer of the humic bog and was the lowest in the oxic layer of the clear-water lake. This distribution pattern is consistent with the availability of the thermodynamically more favorable electron acceptor, i.e., oxygen, between the two layers and the much higher concentrations of HS and Fe in the bog than in the clear-water lake.

It was not surprising to find putative EET genes in relatives of bacteria that are known to be capable of Fe redox reactions and HS reduction in anoxic lake waters. However, finding putative EET genes in taxa not known for EET functions is intriguing. Like many known EET organisms, some of these bacteria (e.g., *Bacteroidetes* and *Verrucomicrobia*) contain multiple sets of putative EET genes. In particular, some *Methylotenera* and *Methylobacter* organisms contain both Cyc2 and putative PCC genes. If these methylotrophs are indeed capable of EET, this might enable insoluble or high-molecular-weight substrates, such as Fe(III) and HS, to be used as an electron acceptor to oxidize the methyl group in methanol and methylamine. Such EET processes, if they occur, might allow methylotrophs to survive in the anoxic layer, and this agrees with the recovery of *Methylotenera* and *Methylobacter* MAGs in the largely anoxic hypolimnion of Trout Bog.

HS, especially its photodegradation products (5), have until now usually been regarded as an electron donor and C source in freshwater lakes and not as an electron acceptor. However, evidence for the role as an electron acceptor was recently documented in another peat bog lake (9). In our study, the electron-accepting capabilities of the Trout Bog epilimnion and hypolimnion water were 0.115 and 0.128 mM, respectively. Notably, these values are an order of magnitude higher than the estimated electron-accepting capacity of Fe (∼0.01 mM) in Trout Bog. Therefore, HS may be a significant, but previously overlooked, source of electron acceptors in the anoxic hypolimnion of this bog system.

**FIG 3:**
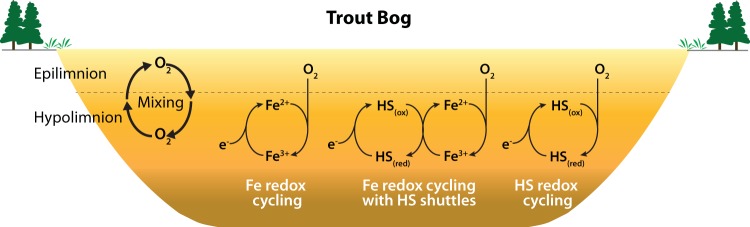
Proposed roles of EET genes in facilitating redox cycling of Fe and HS in Trout Bog. Oxygenation in the hypolimnion through seasonal mixing and more frequent micromixing (such as wind-driven turbulence and convectively derived diurnal oxycline fluctuations) regenerates the electron-accepting capacity of reduced HS and Fe to enable these anaerobic respiration processes sustainable in the hypolimnion.

Due to its high electron-accepting capacity and concentration, HS may play an important role in the redox cycling in Trout Bog. On one hand, HS facilitates Fe redox reactions by shuttling electrons from Fe(III) reducers to Fe(III) in heterotrophic respiration (17). On the other hand, HS may be directly used as an electron acceptor to respire the more labile organic C (Fig. 3). The anaerobic respiration of organic C with both Fe(III) and HS is thermodynamically more favorable than methanogenesis, therefore promoting the transformation of organic C toward CO_2_, not CH_4_. This might lower the overall global-warming potential of greenhouse gas emissions from humic lakes, as CH_4_ is a much more potent greenhouse gas than CO_2_. Because of lake seasonal mixing and more frequent micromixing, such as wind-driven turbulence and convectively derived diurnal oxycline fluctuations (9), reduced HS and Fe can be reoxidized through mixing introduced oxygenation to regenerate their electron-accepting capacity, which makes these anaerobic respiration processes sustainable in the anoxic layer (Fig. 3). In these redox processes, oxygen is the ultimate electron acceptor, and Fe and HS “recharge” the electron-accepting capacity through the reoxidation by oxygen for subsequent use when oxygen becomes unavailable in stratified hypolimnia. Hypothetically, such a recharging process would increase the effective electron-accepting capacity of humic water and shunt more electrons to anaerobic respiration, i.e., in a manner analogous to mediation of lake water C metabolism via cryptic Fe redox cycling (6). Therefore, we hypothesize that HS, as well as Fe, may be a previously overlooked electron acceptor and EET may be an important contribution to pelagic respiration in humic-rich freshwater lakes. Coupled with C metabolism, EET-enabled HS and Fe redox dynamics can significantly influence C cycling and greenhouse gas emission in humic lakes that experience recurrent oxic/anoxic conditions. The overrepresentation of EET genes/organisms potentially involved in HS and Fe redox processes in the humic lake is consistent with this hypothesis, given that the energetic advantage that such organisms can obtain stays marginal when powerful recharge mechanisms at the oxic/anoxic interface are lacking. Yet further combined biogeochemical, hydrodynamic, genomic, and transcriptomic studies are required to test our hypothesis and reveal organisms and genes actually involved *in situ*.

## References

[1] Monteith DT, Stoddard JL, Evans CD, de Wit HA, Forsius M, Høgåsen T, Wilander A, Skjelkvåle BL, Jeffries DS, Vuorenmaa J, Keller B, Kopácek J, Vesely J 2007 Dissolved organic carbon trends resulting from changes in atmospheric deposition chemistry. Nature 450:537. doi:10.1038/nature06316. 18033294

[2] Freeman C, Evans CD, Monteith DT, Reynolds B, Fenner N 2001 Export of organic carbon from peat soils. Nature 412:785. doi:10.1038/35090628. 11518954

[3] Neal C, Lofts S, Evans C, Reynolds B, Tipping E, Neal M 2008 Increasing iron concentrations in UK upland waters. Aquat Geochem 14:263–288. doi:10.1007/s10498-008-9036-1.

[4] Sarkkola S, Nieminen M, Koivusalo H, Lauren A, Kortelainen P, Mattsson T, Palviainen M, Piirainen S, Starr M, Finer L 2013 Iron concentrations are increasing in surface waters from forested headwater catchments in eastern Finland. Sci Total Environ 463-464:683–689. doi:10.1016/j.scitotenv.2013.06.072. 23850658

[5] Bertilsson S, Stefan LJ 1998 Photochemically produced carboxylic acids as substrates for freshwater bacterioplankton. Limnol Oceanogr 43:885–895. doi:10.4319/lo.1998.43.5.0885.

[6] Berg JS, Michellod D, Pjevac P, Martinez-Perez C, Buckner CR, Hach PF, Schubert CJ, Milucka J, Kuypers MM 2016 Intensive cryptic microbial iron cycling in the low iron water column of the meromictic Lake Cadagno. Environ Microbiol 18:5288–5302. doi:10.1111/1462-2920.13587. 27768826

[7] Klupfel L, Piepenbrock A, Kappler A, Sander M 2014 Humic substances as fully regenerable electron acceptors in recurrently anoxic environments. Nature Geosci 7:195–200. doi:10.1038/ngeo2084.

[8] Kappler A, Benz M, Schink B, Brune A 2004 Electron shuttling via humic acids in microbial iron(III) reduction in a freshwater sediment. FEMS Microbiol Ecol 47:85–92. doi:10.1016/S0168-6496(03)00245-9. 19712349

[9] Lau MP, Hupfer M, Grossart HP 2017 Reduction-oxidation cycles of organic matter increase bacterial activity in the pelagic oxycline. Environ Microbiol Rep 9:257–267. doi:10.1111/1758-2229.12526. 28217926

[10] Miller KE, Lai C-T, Friedman ES, Angenent LT, Lipson DA 2015 Methane suppression by iron and humic acids in soils of the Arctic Coastal Plain. Soil Biol Biochem 83:176–183. doi:10.1016/j.soilbio.2015.01.022.

[11] Maizel AC, Li J, Remucal CK 2017 Relationships between dissolved organic matter composition and photochemistry in lakes of diverse trophic status. Environ Sci Technol 51:9624–9632. doi:10.1021/acs.est.7b01270. 28719191 PMC5881397

[12] Bendall ML, Stevens SLR, Chan L-K, Malfatti S, Schwientek P, Tremblay J, Schackwitz W, Martin J, Pati A, Bushnell B, Froula J, Kang D, Tringe SG, Bertilsson S, Moran MA, Shade A, Newton RJ, McMahon KD, Malmstrom RR 2016 Genome-wide selective sweeps and gene-specific sweeps in natural bacterial populations. ISME J 10:1589–1601. doi:10.1038/ismej.2015.241. 26744812 PMC4918448

[13] Linz AM, He S, Stevens SL, Anantharaman K, Rohwer RR, Malmstrom RR, Bertilsson S, McMahon KD 2018 Freshwater carbon and nutrient cycles revealed through reconstructed population genomes. PeerJ 6:e6075. doi:10.7717/peerj.6075. 30581671 PMC6292386

[14] Castelle C, Guiral M, Malarte G, Ledgham F, Leroy G, Brugna M, Giudici-Orticoni MT 2008 A new iron-oxidizing/O_2_-reducing supercomplex spanning both inner and outer membranes, isolated from the extreme acidophile *Acidithiobacillus ferrooxidans*. J Biol Chem 283:25803–25811. doi:10.1074/jbc.M802496200. 18632666 PMC3258861

[15] Mehta T, Coppi MV, Childers SE, Lovley DR 2005 Outer membrane c-type cytochromes required for Fe(III) and Mn(IV) oxide reduction in Geobacter sulfurreducens. Appl Environ Microbiol 71:8634–8641. doi:10.1128/AEM.71.12.8634-8641.2005. 16332857 PMC1317342

[16] Beliaev AS, Saffarini DA 1998 *Shewanella putrefaciens mtrB* encodes an outer membrane protein required for Fe(III) and Mn(IV) reduction. J Bacteriol 180:6292–6297. 9829939 10.1128/jb.180.23.6292-6297.1998 PMC107715

[17] Coates JD, Ellis DJ, Blunt-Harris EL, Gaw CV, Roden EE, Lovley DR 1998 Recovery of humic-reducing bacteria from a diversity of environments. Appl Environ Microbiol 64:1504–1509. 9546186 10.1128/aem.64.4.1504-1509.1998 PMC106177

[18] Voordeckers JW, Kim BC, Izallalen M, Lovley DR 2010 Role of Geobacter sulfurreducens outer surface c-type cytochromes in reduction of soil humic acid and anthraquinone-2,6-disulfonate. Appl Environ Microbiol 76:2371–2375. doi:10.1128/AEM.02250-09. 20154112 PMC2849258

[19] Bucking C, Piepenbrock A, Kappler A, Gescher J 2012 Outer-membrane cytochrome-independent reduction of extracellular electron acceptors in Shewanella oneidensis. Microbiology 158:2144–2157. doi:10.1099/mic.0.058404-0. 22493303

[20] Shyu JB, Lies DP, Newman DK 2002 Protective role of tolC in efflux of the electron shuttle anthraquinone-2,6-disulfonate. J Bacteriol 184:1806–1810. 11872737 10.1128/JB.184.6.1806-1810.2002 PMC134904

[21] Bewley KD, Ellis KE, Firer-Sherwood MA, Elliott SJ 2013 Multi-heme proteins: nature's electronic multi-purpose tool. Biochim Biophys Acta 1827:938–948. doi:10.1016/j.bbabio.2013.03.010. 23558243 PMC3880547

[22] Jiao Y, Newman DK 2007 The *pio* operon is essential for phototrophic Fe(II) oxidation in *Rhodopseudomonas palustris* TIE-1. J Bacteriol 189:1765–1773. doi:10.1128/JB.00776-06. 17189359 PMC1855732

[23] Emerson D, Field EK, Chertkov O, Davenport KW, Goodwin L, Munk C, Nolan M, Woyke T 2013 Comparative genomics of freshwater Fe-oxidizing bacteria: implications for physiology, ecology, and systematics. Front Microbiol 4:254. doi:10.3389/fmicb.2013.00254. 24062729 PMC3770913

[24] Liu Y, Wang Z, Liu J, Levar C, Edwards MJ, Babauta JT, Kennedy DW, Shi Z, Beyenal H, Bond DR, Clarke TA, Butt JN, Richardson DJ, Rosso KM, Zachara JM, Fredrickson JK, Shi L 2014 A trans-outer membrane porin-cytochrome protein complex for extracellular electron transfer by *Geobacter sulfurreducens* PCA. Environ Microbiol Rep 6:776–785. doi:10.1111/1758-2229.12204. 25139405 PMC4282303

[25] He S, Barco RA, Emerson D, Roden EE 2017 Comparative genomic analysis of neutrophilic iron(II) oxidizer genomes for candidate genes in extracellular electron transfer. Front Microbiol 8:1584. doi:10.3389/fmicb.2017.01584. 28871245 PMC5566968

[26] Carlson HK, Iavarone AT, Gorur A, Yeo BS, Tran R, Melnyk RA, Mathies RA, Auer M, Coates JD 2012 Surface multiheme c-type cytochromes from Thermincola potens and implications for respiratory metal reduction by Gram-positive bacteria. Proc Natl Acad Sci U S A 109:1702–1707. doi:10.1073/pnas.1112905109. 22307634 PMC3277152

[27] Skennerton CT, Chourey K, Iyer R, Hettich RL, Tyson GW, Orphan VJ 2017 Methane-fueled syntrophy through extracellular electron transfer: uncovering the genomic traits conserved within diverse bacterial partners of anaerobic methanotrophic archaea. mBio 8:e00530-17. doi:10.1128/mBio.00530-17. 28765215 PMC5539420

