# Supplementary figures and images for: Extracellular Electron Transfer May Be an Overlooked Contribution to Pelagic Respiration in Humic-Rich Freshwater Lakes
Source: mSphere. 2019 Jan 23;4(1):e00436-18. doi: 10.1128/mSphere.00436-18 (PMC6344600; doi:10.1128/mSphere.00436-18)

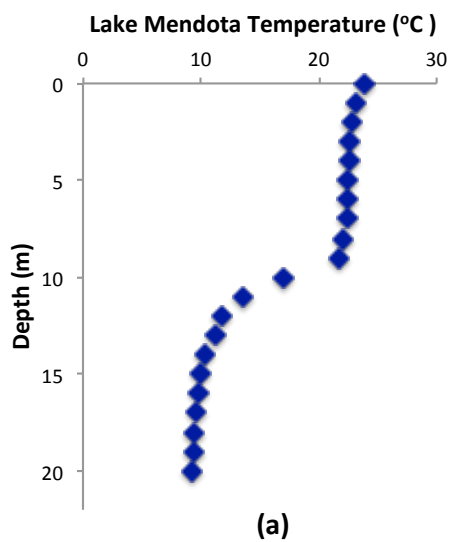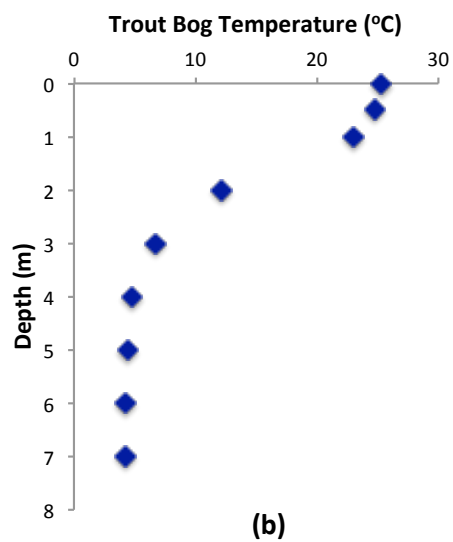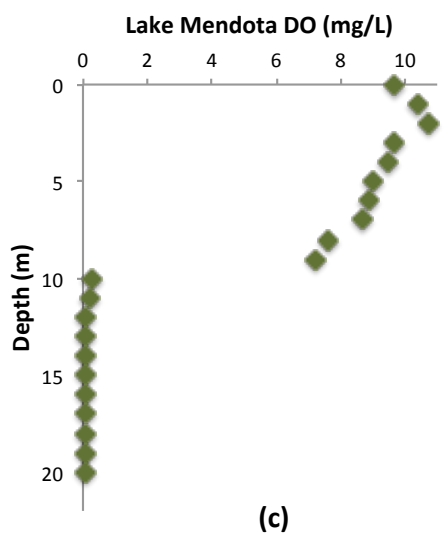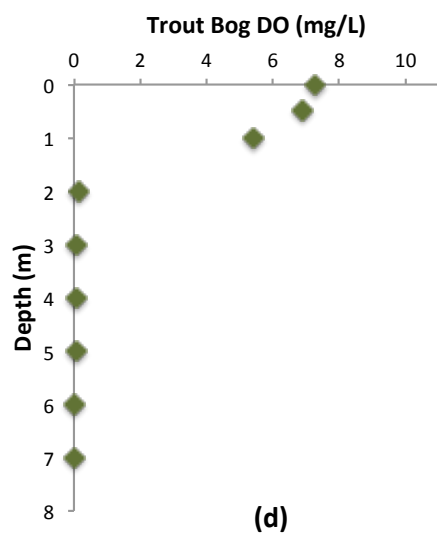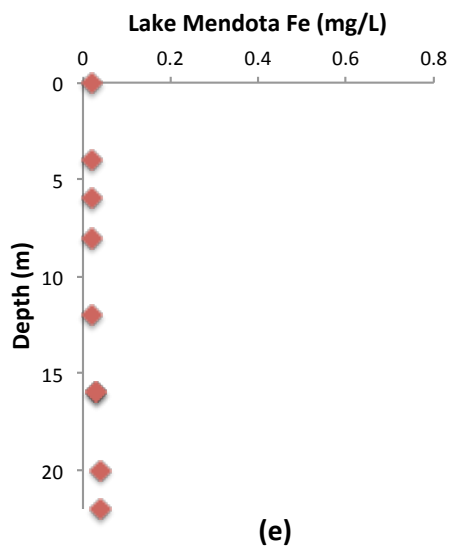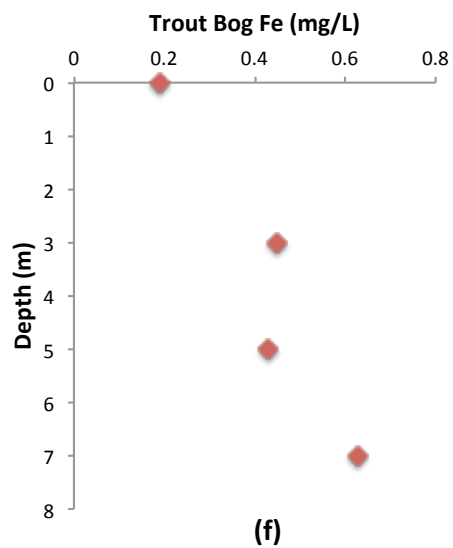

Supplement: FIG S1 [file mSphere.00436-18-sf001.pdf]
