# Supplementary material for: Extracellular Electron Transfer May Be an Overlooked Contribution to Pelagic Respiration in Humic-Rich Freshwater Lakes
Source: mSphere. 2019 Jan 23;4(1):e00436-18. doi: 10.1128/mSphere.00436-18 (PMC6344600; doi:10.1128/mSphere.00436-18)
Supplement: TEXT S1 [file mSphere.00436-18-s0001.docx]

**Supplemental Methods**

**Identification of Cyc2 homologs**

Cyc2 homologs were obtained by BLASTP search using *Acidithiobacillus ferrooxidans* ATCC 53993 Cyc2 sequence (NCBI Accession number YP_002221150) as the reference against our metagenome databases, with an E-value cutoff of 1e-5. Cyc2 is expected to be outer membrane protein that has a relatively conserved N-terminus and a beta-barrel structure in the C-terminus ([1](#_ENREF_1)). Therefore, we checked the predicted cellular location of these Cyc2 homologs using CELLO v.2.5 ([http://cello.life.nctu.edu.tw](http://cello.life.nctu.edu.tw/)) ([2](#_ENREF_2)) and the beta-barrel structure of outer membrane proteins using PRED-TMBB (<http://bioinformatics.biol.uoa.gr//PRED-TMBB>) ([3](#_ENREF_3)). A small number of sequences that were predicted to locate in the cytoplasm or periplasm or proteins without a beta-barrel structure, or sequences too short for accurate prediction were excluded from the Cyc2 list. We also checked the alignment of these Cyc2 homologous sequences and they have conserved N-terminus with most of the conserved residues often found in Cyc2 genes ([4](#_ENREF_4)).

**Identification of outer membrane/extracellular MHC and PCC**

Multiheme cytochrome c (MHC) was identified by the canonical heme-binding motif (CXXCH), and only MHC with at least five heme-binding motifs were retained for further evaluation if they are likely EET genes, which include MHC component of PCC and outer membrane/extracellular MHCs that are not components of PCC. In total, we found 1018 MHCs with at least five heme-binding sites from the three metagenomes. For each of them, we checked their predicted cellular locations using CELLO v.2.5, and genes upstream and downstream of the MHC to see if there is a gene encoding a beta-barrel outer membrane protein that likely forms a porin using PRED-TMBB.

If a predicted periplasmic MHC gene is next to a porin-coding gene, and optionally with an additional predicted extracellular MHC gene nearby, their protein products might be able to form PCC, as in the case of MtrABC in *Shewanella* spp. ([5](#_ENREF_5)), OmbB-OmaB-OmcB in *Geobacter* spp. ([6](#_ENREF_6)), and some other recently proposed PCC systems ([4](#_ENREF_4)). However, if the MHC is on a very short contig or at the end of a long contig, the associated porin-coding gene may be missing due to genome incompleteness. For these MHCs, we still identify the MHC as a putative PCC component, if its homologous MHC (with >60% protein identity) is a putative PCC component. In such a case, the homologous MHC gene locus tag is also listed in Table S2 for reference. The normalized abundance of porin-associated MHCs identified based on homology accounted for about 16% of all porin-associated MHCs.

For MHCs that are not associated with a porin-coding gene by checking gene neighborhood or sequence homology described above, they are not likely components of PCC. For such MHCs, if they are predicted outer membrane/extracellular proteins, they might be able to involve in EET as the outer membrane MHCs in *Geobacter* spp. ([7](#_ENREF_7), [8](#_ENREF_8)) and the cell wall-associated MHCs in Gram positive Fe(III)- and AQDS-reducing firmicutes ([9](#_ENREF_9)).

**Calculation of normalized abundance**

Metagenomes from the three combined assemblies were annotated at IMG (<https://img.jgi.doe.gov/m>), with the IMG IDs of 3300002835, 3300000439, and 3300000553 for the ME, TE and TH metagenome, respectively. The abundance of an individual gene in the metagenome was estimated using its average per-base coverage, which indicates how many times a base is sequenced and was determined by mapping metagenome reads to assembled contigs. For the three combined metagenomes, such coverage estimate was available at the IMG database as “read depth”, and was referred to as “coverage-weighted abundance” in this study to reflect the abundance of the population that possesses this gene. To compare gene abundance among different metagenomes, the relative abundance of a gene was estimated by normalizing (i.e. dividing) the coverage-weighted abundance of this gene by the average of coverage-weighted abundance of single-copy conserved bacterial housekeeping genes ([10](#_ENREF_10)) in the same metagenome, and this estimate was referred to as “normalized abundance”.

**Determination of lake water EAC**

The electron accepting capacity (EAC) of HS in the epilimnion and hypolimnion water of Trout Bog was determined according to the methods first described by Kappler et al. ([11](#_ENREF_11)). Briefly, duplicate 10-mL lake water samples were transferred to anaerobic pressure tubes (Belco Glass Co.). One set of tubes was flushed with 100% N_2_, and another set with 100% H_2_ in the presence of five palladium-coated silica pellets (Sigma-Aldrich). The tubes were incubated horizontally on a shaker (100 rpm) overnight, after which the electron donating capacity of lake water DOC was analyzed by the Fe(III)-based electron shuttling assay ([12](#_ENREF_12)) as described in Roden et al. ([13](#_ENREF_13)). The EAC of HS was calculated from the difference between reducing equivalents in the H_2_- vs. N_2_-flushed lake water. The amount of reducing equivalents in the N_2_-flushed lake water was insignificant compared to that in the H_2_-flushed samples.

**Supplemental Reference**

1. **White GF, Edwards MJ, Gomez-Perez L, Richardson DJ, Butt JN, Clarke TA**. 2016. Mechanisms of Bacterial Extracellular Electron Exchange. Adv Microb Physiol **68**:87-138.

2. **Yu CS, Chen YC, Lu CH, Hwang JK**. 2006. Prediction of protein subcellular localization. Proteins **64**:643-51.

3. **Bagos PG, Liakopoulos TD, Spyropoulos IC, Hamodrakas SJ**. 2004. PRED-TMBB: a web server for predicting the topology of beta-barrel outer membrane proteins. Nucleic Acids Res **32**:W400-4.

4. **He S, Barco RA, Emerson D, Roden EE**. 2017. Comparative Genomic Analysis of Neutrophilic Iron(II) Oxidizer Genomes for Candidate Genes in Extracellular Electron Transfer. Frontiers in Microbiology **8**.

5. **Beliaev AS, Saffarini DA**. 1998. *Shewanella putrefaciens* *mtrB* encodes an outer membrane protein required for Fe(III) and Mn(IV) reduction. J Bacteriol **180**:6292-7.

6. **Liu Y, Wang Z, Liu J, Levar C, Edwards MJ, Babauta JT, Kennedy DW, Shi Z, Beyenal H, Bond DR, Clarke TA, Butt JN, Richardson DJ, Rosso KM, Zachara JM, Fredrickson JK, Shi L**. 2014. A trans-outer membrane porin-cytochrome protein complex for extracellular electron transfer by *Geobacter sulfurreducens* PCA. Environ Microbiol Rep **6**:776-85.

7. **Leang C, Coppi MV, Lovley DR**. 2003. OmcB, a c-type polyheme cytochrome, involved in Fe(III) reduction in Geobacter sulfurreducens. J Bacteriol **185**:2096-103.

8. **Mehta T, Coppi MV, Childers SE, Lovley DR**. 2005. Outer membrane c-type cytochromes required for Fe(III) and Mn(IV) oxide reduction in *Geobacter sulfurreducens*. Appl Environ Microbiol **71**:8634-41.

9. **Carlson HK, Iavarone AT, Gorur A, Yeo BS, Tran R, Melnyk RA, Mathies RA, Auer M, Coates JD**. 2012. Surface multiheme c-type cytochromes from Thermincola potens and implications for respiratory metal reduction by Gram-positive bacteria. Proceedings of the National Academy of Sciences **109**:1702-1707.

10. **Rinke C, Schwientek P, Sczyrba A, Ivanova NN, Anderson IJ, Cheng J-F, Darling A, Malfatti S, Swan BK, Gies EA, Dodsworth JA, Hedlund BP, Tsiamis G, Sievert SM, Liu W-T, Eisen JA, Hallam SJ, Kyrpides NC, Stepanauskas R, Rubin EM, Hugenholtz P, Woyke T**. 2013. Insights into the phylogeny and coding potential of microbial dark matter. Nature **499**:431-437.

11. **Kappler A, Benz M, Schink B, Brune A**. 2004. Electron shuttling via humic acids in microbial iron(III) reduction in a freshwater sediment. FEMS Microbiol Ecol **47**:85-92.

12. **Lovley DR, Coates JD, Blunt-Harris EL, Phillips EJP, Woodward JC**. 1996. Humic substances as electron acceptors for microbial respiration. Nature **382**:445.

13. **Roden EE, Kappler A, Bauer I, Jiang J, Paul A, Stoesser R, Konishi H, Xu H**. 2010. Extracellular electron transfer through microbial reduction of solid-phase humic substances. Nature geoscience **3**:417-421.
